# The Endo-siRNA Pathway Is Essential for Robust Development of the *Drosophila* Embryo

**DOI:** 10.1371/journal.pone.0007576

**Published:** 2009-10-23

**Authors:** Elena M. Lucchetta, Richard W. Carthew, Rustem F. Ismagilov

**Affiliations:** 1 Department of Chemistry and Institute for Biophysical Dynamics, The University of Chicago, Chicago, Illinois, United States of America; 2 Department of Biochemistry, Molecular Biology, and Cell Biology, Northwestern University, Evanston, Illinois, United States of America; Katholieke Universiteit Leuven, Belgium

## Abstract

**Background:**

Robustness to natural temperature fluctuations is critical to proper development in embryos and to cellular functions in adult organisms. However, mechanisms and pathways which govern temperature compensation remain largely unknown beyond circadian rhythms. Pathways which ensure robustness against temperature fluctuations may appear to be nonessential under favorable, uniform environmental conditions used in conventional laboratory experiments where there is little variation for which to compensate. The endo-siRNA pathway, which produces small double-stranded RNAs in *Drosophila*, appears to be nonessential for robust development of the embryo under ambient uniform temperature and to be necessary only for viral defense. Embryos lacking a functional endo-siRNA pathway develop into phenotypically normal adults. However, we hypothesized that small RNAs may regulate the embryo's response to temperature, as a ribonucleoprotein complex has been previously shown to mediate mammalian cell response to heat shock.

**Principal Findings:**

Here, we show that the genes DICER-2 and ARGONAUTE2, which code for integral protein components of the endo-siRNA pathway, are essential for robust development and temperature compensation in the *Drosophila* embryo when exposed to temperature perturbations. The regulatory functions of DICER-2 and ARGONAUTE2 were uncovered by using microfluidics to expose developing *Drosophila* embryos to a temperature step, in which each half of the embryo develops at a different temperature through developmental cycle 14. Under this temperature perturbation, *dicer-2* or *argonaute2* embryos displayed abnormal segmentation. The abnormalities in segmentation are presumably due to the inability of the embryo to compensate for temperature-induced differences in rate of development and to coordinate developmental timing in the anterior and posterior halves. A deregulation of the length of nuclear division cycles 10–14 is also observed in *dicer-2* embryos at high temperatures.

**Conclusions:**

Results presented herein uncover a novel function of the endo-siRNA pathway in temperature compensation and cell cycle regulation, and we hypothesize that the endo-siRNA pathway may regulate the degradation of maternal cell cycle regulators. Endo-siRNAs may have a more general role buffering against environmental perturbations in other organisms.

## Introduction

“There is no greater biological question of greater moment than the means by which the individual cell activities are coordinated, and the organic unity of the body maintained” [1]. In the developing embryo, the coordination of individual cell activities, or robustness, under environmental fluctuations such as temperature is critical in forming the adult organism. Although robustness to temperature fluctuations is critical to proper development, the pathways and molecular mechanisms that govern robustness have long remained perplexing [2]–[4]. Mechanisms of robustness against temperature fluctuations have been largely unexplored outside of circadian rhythms [5], [6].

In developing embryos such as those of *Drosophila melanogaster*, a hierarchy of precise protein expression guides the growth of the embryo in space and time at a large range of temperatures. These patterns separate the embryo into well-defined segments that form the adult body plan. In *Drosophila*, the earliest segmented patterns of protein expression appear in the embryo while it is a syncytium, i.e. concurrent with rapid nuclear divisions in a common cytoplasm. Later, the syncitial blastoderm cellularizes into thousands of cells, each cell inheriting the pattern of gene expression expressed by its nucleus during cellularization. The lack of cellular compartmentalization in developing *Drosophila* embryos makes the maintenance of robustness and coordination of cellular processes at a large range of temperatures during early development particularly puzzling.

The pathways involved in maintaining robustness to temperature fluctuations may appear to be nonessential under favorable, uniform environmental conditions such as those used in conventional laboratory experiments. However, the importance of these pathways can be revealed under controlled environmental perturbations [7]–[9]. We previously developed a microfluidic platform that can be used to precisely control and perturb the environment around a syncitial *Drosophila* embryo by maintaining each half of the embryo at a specific and different temperature [10], [11], i.e. creating a temperature step across the body of the embryo. Early embryonic events, such as nuclear divisions, occur asynchronously in such temperature perturbations, and early segmentation events are also asynchronous. Remarkably, both the density of nuclei across the embryo [12] and segmented gene expression [10] become stabilized by cycle 14, and embryos develop into normal animals. Although this paradigm reveals the degree of robustness to temperature inherent in embryonic segmentation and development, the pathways responsible for temperature compensation have remained unclear.

**Figure 1:**
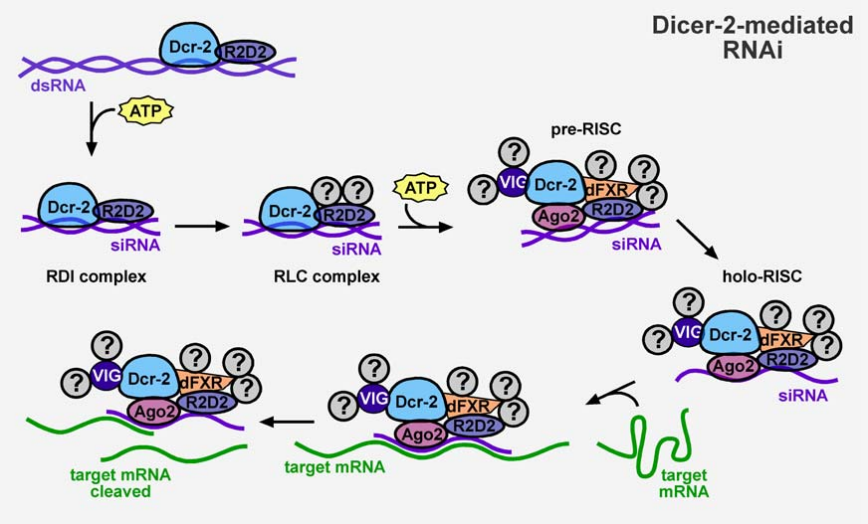
siRNA silencing pathway initiated by Dicer-2 (Dcr-2). Dcr-2 and R2D2 process dsRNAs into small interfering (siRNAs) in an ATP-dependent mechanism. The double-stranded siRNA is then assembled in a pre-RISC complex containing Argonaute2 (Ago2) prior to forming the holo-RISC complex, which contains only the guide siRNA strand and facilitates cleavage of target mRNA.

It has been speculated that microRNAs (miRNAs) might be used to build robustness within biological networks [13]. There are examples of individual miRNAs that impart robustness to developmental decisions in various organisms [14]–[17]. Moreover, bioinformatic analysis suggests that 45–70% of mammalian miRNAs act in feedback and feedforward network motifs [18], which build robustness into networks. miRNAs are related to small interfering RNAs (siRNAs) in many respects. Both types of RNAs are produced from double-stranded RNA (dsRNA) precursors by post-transcriptional processing by the Dicer (Dcr) ribonuclease [19]. Both types of RNAs are associated with Argonaute (Ago) proteins to form ribonucleoprotein effector complexes. Both types of RNAs repress gene expression by base-pairing with messenger RNAs of complementary sequence. Despite these similarities, there are several differences between miRNAs and siRNAs. While miRNAs are processed from short imperfect hairpin RNAs, the dsRNA precursors for siRNAs are generally perfect duplexes or extended hairpins. Animal miRNAs typically repress mRNA translation by imperfect pairing to messages, while siRNAs cause mRNA degradation by a perfect pairing mechanism. Moreover, in some species different Dcr and Ago proteins are specific for particular miRNAs or siRNAs. For example, *Drosophila* Dicer-1 (Dcr-1) processes miRNAs, whereas Dicer-2 (Dcr-2) processes siRNAs [20] (Figure 1). Ago1 loads miRNAs, whereas Ago2 loads siRNAs. Thus, the two types of RNAs can be distinguished by a number of features.

Although miRNAs have been implicated in building robustness, they are also important for major developmental events affecting cell differentiation, division, morphogenesis, and apoptosis [21]. This broad range of functions for miRNAs might be due to their sheer numbers (at least in the hundreds) and their broad specificity. In contrast, siRNAs appear dispensable for major developmental events under uniform laboratory conditions. This is most simply evident in *Drosophila*, where mutations in DCR-2 and AGO2 specifically impair the siRNA pathway but have negligible effect on fly development–mutants are viable and fertile [20], [22]. A few weak defects have been described for *ago2* embryos but these defects do not impair the outcome of embryogenesis [23]. Instead, the *Drosophila* siRNA pathway appears to be critical only for innate immunity against viral infection. Viral RNAs are processed by Dcr-2 into siRNAs that repress viral gene expression, and this host mechanism attenuates virus replication and pathogenicity [24]–[26].

These findings might suggest that siRNAs are derived only from exogenous RNA sources as a defensive response against foreign agents. However, it is becoming clear that siRNAs are also derived from endogenous RNA sources. These endo-siRNAs were first described in the nematode *Caenorhabditis elegans*, and were implicated in silencing expression of transposable elements within its genome [27], [28]. More recently, endo-siRNAs have been discovered to be prevalent in *Drosophila* at many stages of development and in distinct tissues [29]–[32]. Genetic experiments demonstrate that Dcr-2 and Ago2 are required for activity of endo-siRNAs. As in *C. elegans, Drosophila* endo-siRNAs corresponding to transposable elements have been found, and they appear to repress these elements. However, many endo-siRNAs have also been found corresponding to various endogenous fly genes, prompting the question as to their functions in *Drosophila*.

Several lines of evidence prompted us to hypothesize that the endo-siRNA pathway may have a role in generating robustness during *Drosophila* embryonic development. Previously, we determined a period of time critical to robust development of embryos exposed to a temperature step, the orientation of which was changed for a 35 minute window [10]. The critical time identified was from 65 to 100 minutes of development, during which nuclear division cycles are very rapid and may only suppsort the synthesis of short zygotic transcripts [33]. Additionally, *staufen^HL^* mutant embryos displayed a decrease in robust development [34], which could be due to another double-stranded RNA binding function of Staufen apart from *bicoid* or *oskar*.

To test our hypothesis that the endo-siRNA pathway is essential for maintaining robustness to temperature fluctuations, we used microfluidics to expose developing *Drosophila* embryos to a temperature step. We uncover a novel function of the endo-siRNA pathway in maintaining robustness to temperature fluctuations in the developing embryo, and show that the endo-siRNA pathway is essential to development under environmental perturbations. This result could shed light on the role of endo-siRNA silencing in robust development among other organisms. In addition, this work demonstrates the usefulness of controlled environmental perturbations to uncover pathways essential for generating robustness.

## Results

### Segmentation is Normal in *dcr-2* and *ago2* Mutant Embryos in Uniform Temperature Environments

Core components of the endo-siRNA pathway are produced and are potentially active in the *Drosophila* germline [35]. Moreover, these components are maternally contributed to the early zygotic embryo [20], [22]. Therefore, to perform our experiments studying early embryogenesis, we generated mutant embryos that are missing both maternal and zygotic expression of endo-siRNA pathway components. This strategy was complicated by the fact that homozygous null *dcr-2* adults show a considerably enhanced susceptibility to viral infection [24], [25]. We wanted to ensure that any effects on embryogenesis that we observed were not due to stresses imparted through viral infection, either to the mother or to the embryo. To obviate this possibility, we prepared null *dcr-2* homozygous embryos from heterozygous wild type (WT) mothers by using the FLP/FRT system to generate homozygous mutant germ cells within these mothers [20], [36]. Such heterozygous females exhibit normal levels of viral infection compared to WT females, as tested by qPCR assays for a variety of *Drosophila* viruses (Marques, J. and Carthew, R.W., unpublished data). The *dcr-2* heterozygous females were crossed to heterozygous mutant males to generate embryos that were missing both maternal and zygotic contributions of the DCR-2 gene. qPCR assays confirmed that there was no increase of the viral load in these embryos relative to controls (Marques, J. and Carthew, R.W., unpublished data).

**Figure 2:**
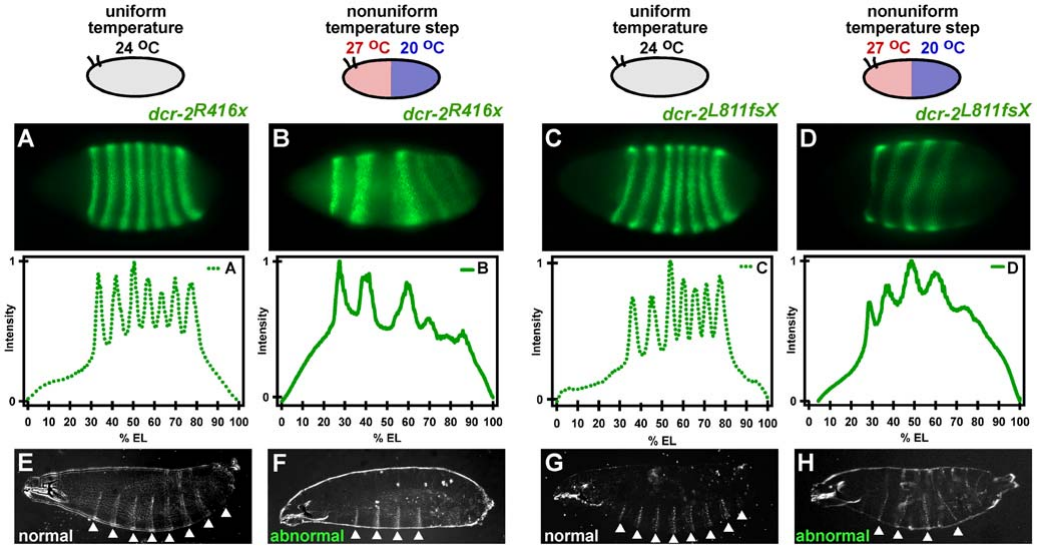
*dcr-2* is required for robust development of segmental patterning in *Drosophila*. (A–D) Eve expression. (A,C) *dcr-2^R416x^* and *dcr-2^L811fsX^* embryos developed at uniform temperature (24°C) have normal Eve stripe positions and express all seven Eve stripes. One of four embryos is shown for *dcr-2^R416x^* and one of three embryos is shown for *dcr-2^L811fsX^* mutants. (B,D) *dcr-2^R416x^* and *dcr-2^L811fsX^* embryos developed in a temperature step have an abnormal number of Eve stripes and abnormal stripe positions. One of seven embryos is shown for *dcr-2^R416x^* and one of four embryos is shown for *dcr-2^L811fsX^* mutants. (E–H) Larval cuticle preparations. (E,G) *dcr-2^R416x^* and *dcr-2^L811fsX^* larvae developed at uniform 24°C have a normal segmentation pattern of denticle belts (arrows). One of five larvae is shown for both *dcr-2^R416x^* and *dcr-2^L811fsX^* mutants. (F,H) *dcr-2^R416x^* and *dcr-2^L811fsX^* larvae that were exposed to the temperature step for the first 200 minutes of development and then allowed to reach larval stage at uniform 24°C have missing or abnormally positioned denticle belts (arrows). One of five larvae is shown for *dcr-2^R416x^* and one of three larvae is shown for *dcr-2^L811fsX^* mutants.

We wished to determine if the loss of DCR-2 results in defects in early segmentation under uniform temperature conditions. We examined this by visualizing the expression profile of the Even-skipped (Eve) protein [37], an early marker of embryonic segmentation (Figure S1). Normal Eve expression is manifested by seven precisely positioned stripes along the antero-posterior axis of the embryo [37]. We found that *dcr-2^R416x^* (Figure 2A) and *dcr-2^L811fsX^* (Figure 2C) embryos displayed normal Eve expression patterns at a uniform temperature of 24°C, as well as at uniform temperatures of 20°C and 27°C (Figure S1A, C–D, F). These mutant embryos subsequently developed into properly segmented larvae having eight abdominal segments (Figure 2E, H and Figure S2A, C). Additionally, *dcr-2^R416x^* embryos displayed normal Eve expression when developing at alternating uniform temperatures, where the temperature was alternated between 20°C and 27°C every 15 or every 30 minutes (data not shown). Similar results were observed in homozygous *ago2* embryos and larvae (Figure S3A). We conclude from these experiments that DCR-2 and AGO2 appear to be nonessential for early embryonic segmentation under uniform temperature conditions.

### Segmentation is Abnormal in *dcr-2* and *ago2* Embryos Exposed to a Temperature Step

Next, we wanted to determine if mutation of the DCR-2 gene results in defects in early segmentation when embryos are exposed to the environmental perturbation of a temperature step. We used a microfluidic device that we had previously developed and characterized [10], [11]. In this device, each half of a live, developing embryo is maintained at a different controlled temperature by two streams of fluid flowing laminarly side by side. In the experiments described below, embryos were allowed to develop in a temperature step with their anterior half at 27°C and their posterior half at 20°C for the first 200 minutes of development, until the cellular blastoderm stage. During the course of these 200 minutes, an anterior and a posterior organizing center, established in the oocyte, normally interact through a cascade of segmentation patterning genes to form the fourteen segments that comprise the fly. Under these temperature step conditions, the anterior and the posterior organizing centers trigger the cascade under very different conditions. The Bicoid gradient is highly abnormal in WT embryos exposed to a temperature step [12], and nuclei divide faster in the warm half of the embryo, creating a difference in nuclear density between the two halves of the embryo [10], [12]. However, the segmentation gene network is highly robust and compensates for any variation that is exerted by the temperature step such that WT embryos correct for differences in nuclear densities and become normally segmented [10] during cycle 14.

**Figure 3:**
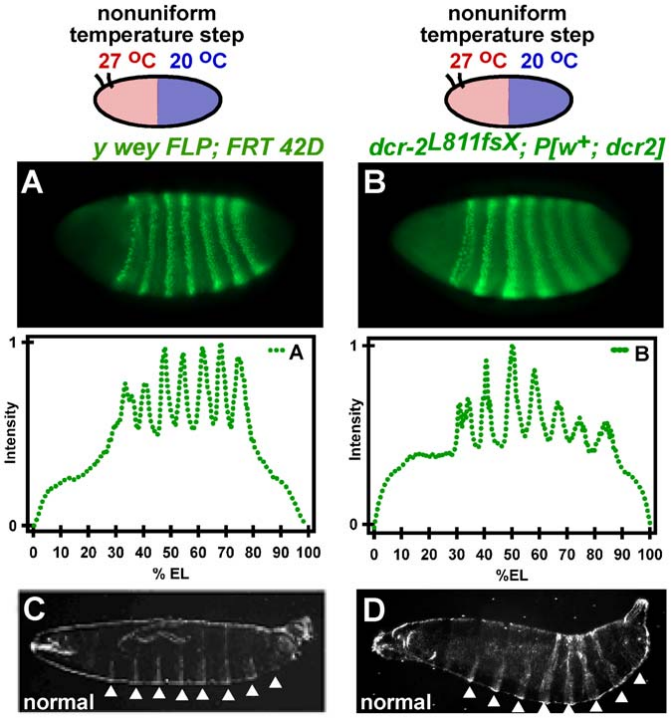
*y w eyFLP; FRT 42D* embryos and *dcr-2^L811fsX^*; *P[w^+^,* DCR-2*]* embryos develop normally in a temperature step, confirming that the abnormalities in segmentation are due to the mutations in DCR-2. (A) A *y w eyFLP; FRT 42D* embryo developed in a temperature step has normal Eve stripe expression and positioning. One of five embryos is shown. (B) A *dcr-2^L811fsX^*; *P[w^+^,* DCR-2*]* embryo shows almost normal Eve patterning. One of three embryos is shown. (C) A *y w eyFLP; FRT 42D* larva that was exposed to the temperature step for the first 200 minutes of embryogenesis had eight abdominal segments (arrows). One of three larvae is shown. (D) A *dcr-2^L811fsX^*; *P[w^+^,* DCR-2*]* larva that was exposed to the temperature step for the first 200 minutes of embryogenesis has almost normal abdominal segments (arrows). One of eight larvae is shown (refer to Figure S4 for additional larvae).

We initially tested that the genetic background was not a complicating factor, exposing both *y w eyFLP; FRT 42D* embryos, and *dcr-2* mutant embryos carrying a rescue DCR-2 transgene to the temperature step. *y w eyFLP; FRT 42D* embryos develop normally in a temperature step–embryos expressed all seven Eve stripes normaly (Figure 3A) and develop into normally segmented larvae after being removed from the temperature step (Figure 3C). Likewise, transgenically rescued embryos compensated for the temperature step and expressed all seven Eve stripes normally (Figure 3B) and developed into normally segmented larvae after being removed from the temperature step (Figure 3D and Figure S4).

We next tested *dcr-2* mutant embryos that did not carry the rescue DCR-2 transgene. Surprisingly, these embryos were unable to compensate for the perturbation of a temperature step (Figure 2B, D). In *dcr-2^R416x^* embryos, between three and five Eve stripes were expressed. In *dcr-2^L811fsX^* embryos, between four and six Eve stripes were expressed. These results indicate that the *dcr-2* embryos lost robust expression and positioning of Eve expression domains.

Embryos with extra copies of maternal factors have been shown to compensate for early segmentation defects prior to reaching larval stage [38]. To determine if *dcr-2* mutant embryos exposed to a temperature step could nevertheless correct for the early abnormalities in segmentation, exoskeletal cuticles were prepared from larvae that had developed in a temperature step for the first 200 minutes of embryogenesis, and then further developed at a uniform temperature of 24°C. Larval cuticles from both *dcr-2^R416x^* (Figure 2F) and *dcr-2^L811fsX^* (Figure 2H) displayed abnormal segmentation. Four out of five *dcr-2^R416x^* larvae had between four and six abdominal segments bearing denticle belts rather than the normal eight segments (Figure 2F and Figure S2B). All three *dcr-2^L811fsX^* larvae had only one to four segments (Figure 2H, and Figure S2D). The loss of segments seen in the mutant larvae is consistent with loss of Eve expression domains seen in *dcr-2* embryos. Thus, the segmentation gene network in *dcr-2* embryos is sensitive to environmental perturbation, resulting in partial loss of segmentation gene expression.

The effect of the loss of DCR-2 in the temperature step is quite specific for segmentation. Segmentation occurs independently of establishment of the body axes [39]. Other gene networks are critical for forming the anteroposterior and dorsoventral axes, though the anteroposterior determinants profoundly influence action of the segmentation network. The *dcr-2* embryos exhibited normal or near-normal body axis organization, as seen by the larval cuticle patterns. Head and tail structures were formed correctly, although tail structures were underdeveloped. Moreover, the *dcr-2* embryos exhibited normal dorsal and ventral identities. These observations argue that null *dcr-2* mutations do not have a general and catastrophic effect on embryonic patterning.

**Figure 4:**
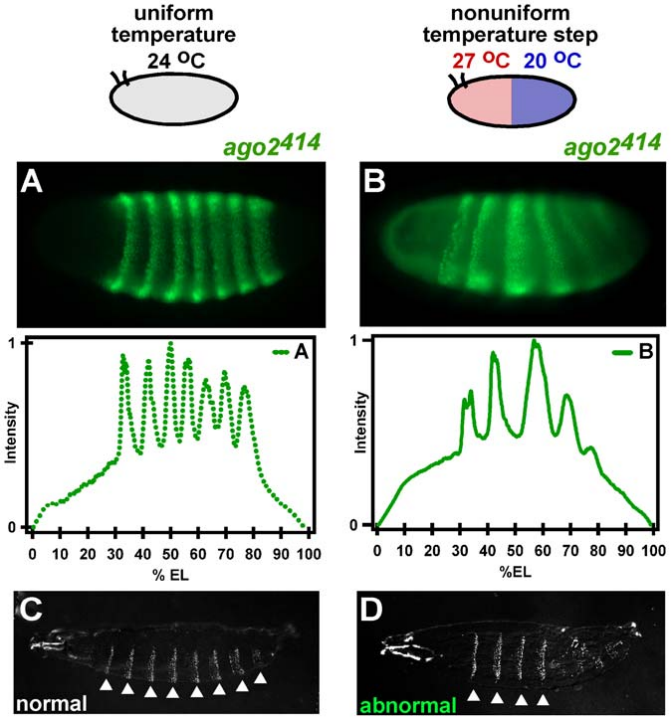
AGO-2 is required for robust segmental patterning in *Drosophila*. (A) An *ago2^414^* embryo developed at uniform temperature (24°C) has normal Eve stripe positions and expresses all seven Eve stripes. One of three embryos is shown. (B) An *ago-2^414^* embryo developed in a temperature step has abnormal expression and positioning of Eve stripes. One of four embryos is shown. (C) *ago2^414^* larvae developed at uniform 24°C have a normal segmentation pattern of denticle belts (arrows). One of four larvae is shown. (D) The cuticle of an *ago2^414^* larva that was exposed to the temperature step for the first 200 minutes of embryogenesis and then allowed to reach larval stage at uniform 24°C has four abdominal segments (arrows). One of three larvae is shown (refer to Figure S3 for additional larvae).

To confirm that the endo-siRNA pathway is critical for robust segmentation and development, we determined the effect of the temperature step on null *ago2^414^* embryos. *ago2^414^* embryos developed normally under uniform temperature conditions (Figure 4A, C), but they displayed abnormal Eve expression when developing in a temperature step with the anterior half at 27°C and the posterior half at 20°C for the first 200 minutes of development (Figure 4B, D). Between four and six Eve stripes were present, with only one out of four embryos showing all seven Eve stripes. Larval cuticles prepared from *ago2^414^* embryos that had developed in a temperature step for the first 200 minutes of embryogenesis were also abnormal (Figure 4D and Figure S3B). Most *ago2^414^* larvae had between four and six abdominal segments, rather than the normal eight segments (Figure S3B). Most mutant larvae exhibited normal head and tail formation, with underdeveloped tail structures, as well as normal dorsoventral patterning, consistent with the *dcr-2* results. Since the *ago2^414^* embryos displayed similar patterning defects to *dcr-2* embryos, we conclude that the endo-siRNA pathway is critical for robustness to temperature during development of the embryo and, therefore, that the mechanism responsible for maintaining robustness of early development is dependent upon endogenous siRNAs. Since virus levels in embryos are not affected by loss of the endo-siRNA pathway under our experimental conditions (Marques, J. and Carthew, R.W., unpublished data), it suggests that the poor robustness of *dcr-2* embryos is not due to their lack of ability to get rid of exogenous viral siRNAs.

### *dcr-2* Embryos Exposed to the Temperature Step Presumably Cannot Compensate for Differences in Rate of Development between the Warm Anterior Half and Cool Posterior Half

**Figure 5:**
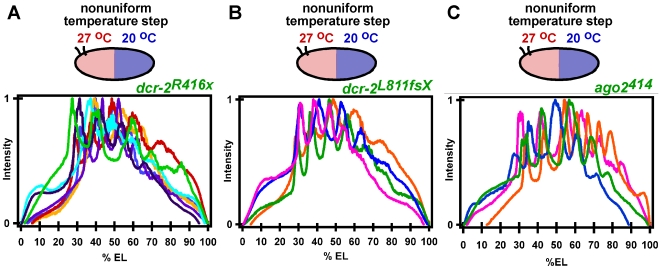
The *dcr-2^R416x^*, *dcr-2^L811fsX^*, and *ago2^414^* mutations cause enhanced variability in Eve stripe expression and position. (A) Intensity profile of Eve expression as a function of embryo length in seven *dcr-2^R416x^* embryos exposed to a temperature step. (B) Intensity profile of Eve expression as a function of embryo length in four *dcr-2^L811fsX^* mutant embryos exposed to a temperature step. (C) Intensity profile of Eve expression as a function of embryo length in four *ago2^414^* embryos exposed to a temperature step.

In addition to the number of Eve stripes, the position of the expressed Eve stripes was also abnormal, varying significantly along the length of the embryo in *dcr-2^R416x^* (Figure 5A), *dcr-2^L811fsX^* (Figure 5B), and *ago2^414^* (Figure 5C) mutant embryos, with posterior Eve expression largely missing.

**Figure 6:**
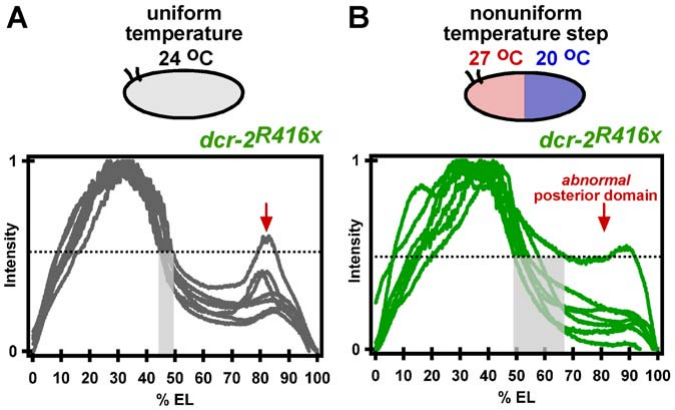
Expression pattern of Hunchback (Hb) in *dcr-2^R416x^* embryos developed at uniform temperature and in a temperature step with anterior at 27°C and posterior at 20°C. (A) *dcr-2^R416x^* embryos developed at uniform 24°C had normal expression of Hb. Seven embryos are plotted. (B) *dcr-2^R416x^* embryos developed in a temperature step with anterior at 27°C and posterior at 20°C displayed slightly more variability in the position of the Hb border. Seven embryos are plotted.

We hypothesized that the missing posterior Eve expression and accompanying loss of posterior larval segments could either be an effect of direct regulation of Eve by the endo-siRNA pathway, or an indirect phenotype caused by an inability to correct for differences in developmental time in the two halves of the embryo, which are at different temperatures. To distinguish between the two mechanisms, we determined the effect of the temperature step in *dicer-2* embryos on the expression of a gap gene which has both anterior and posterior expression domains. If the abnormalities in segmentation seen in *dicer-2* embryos exposed to the temperature step are due to an inability to correct for difference in developmental rates between the two halves of the embryo, we expected to observe a normal anterior domain of gap gene expression, and an underdeveloped or missing posterior domain of gap gene expression. We examined the expression of the protein Hunchback (Hb) [40]–[42], which is one of the first zygotic proteins expressed upstream of Eve in the segmentation pathway and serves an early marker of embryonic segmentation [43]. Normal Hb expression is manifested by both an anterior and posterior domain. Hb expression in the anterior half of the embryo has a sharp boundary at or near the middle of the embryo. This boundary is precisely positioned with little variation between embryos - the point at which the Hb protein profile crosses 50% maximum concentration is between 45 and 50% of the embryonic length (EL) [10], [34]. A stripe of Hb expression is also normally formed in the posterior of the embryo. We found that the precision and position of the Hb boundary is normal in *dcr-2^R416x^* embryos developed under uniform temperature of 24°C (Figure 6A) and that these embryos also express the posterior Hb domain.

In *dcr-2^R416x^* embryos exposed to the temperature step with the anterior half at 27°C and the posterior half at 20°C, the boundary of anterior Hb expression was only slightly abnormal. The boundary position was largely precise, with only one of seven embryos displaying an anterior Hb domain boundary shifted towards the posterior (67% EL) (p = 0.013 comparing the % EL at which the anterior Hb domain boundary reaches half maximum intensity in Figure 6A–B). Notably, a more severe defect was observed in the posterior peak of Hb expression (Figure 6B), that is normally positioned at 80 to 88% of EL [34]. This expression peak is formed under the direction of the terminal gap genes and terminal signaling [44]. In *dcr-2* embryos exposed to the temperature step, the posterior Hb expression was nearly absent in most embryos. However, in one of seven embryos, the posterior Hb domain was present but positioned more posteriorly.

**Figure 7:**
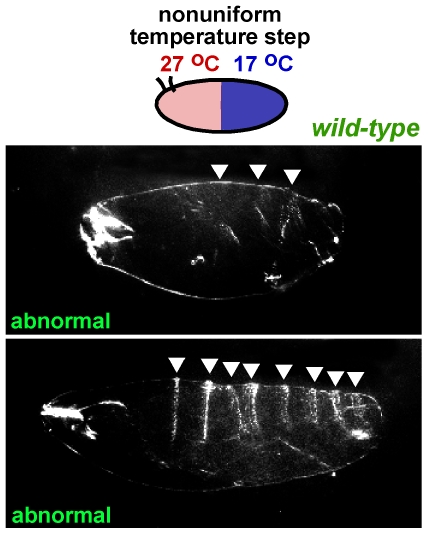
Wild-type (WT) embryos exposed to a larger temperature step display abnormal segmentation. WT embryos developing in a temperature step with anterior at 27°C and posterior at 17°C for the first 200 minutes of development display the same larval phenotype as *dcr-2* mutant embryos developing in a temperature step with anterior at 27°C and posterior at 20°C for the first 200 minutes of development. All larvae are shown.

The missing posterior expression of both Hb and Eve in embryos exposed to the temperature step suggests that embryos lacking an intact endo-siRNA pathway may lack the ability of WT embryos [8] to compensate for differences in the rate of development between the warm and cool halves. If *dcr-2* embryos are unable to compensate for differences in the rate of development between the two halves at different temperatures, we would predict that WT embryos exposed to a larger temperature step (which would induce a greater difference in rate of development) would show a similar phenotype to *dcr-2* embryos exposed to a smaller temperature step. We previously found that a greater difference in rates of development between the anterior and posterior halves could be induced by increasing the temperature difference between the two laminar flows [10]. To test our hypothesis, WT embryos were exposed to a larger temperature step, with their anterior half at 27°C and their posterior half at 17°C for the first 200 minutes of development, and then allowed to reach larval stage at uniform 24°C. As shown in Figure 7, such embryos displayed abnormal segmentation. The phenotypes observed were similar to those of *dcr-2* and *ago2* embryos exposed to a smaller temperature step. These results imply that *dcr-2* and *ago2* embryos display abnormal segmental patterning due to a decreased tolerance to differences in developmental rates.

### The Cell Cycle is Abnormal in *dcr-2* Embryos at High Uniform Temperatures

**Figure 8:**
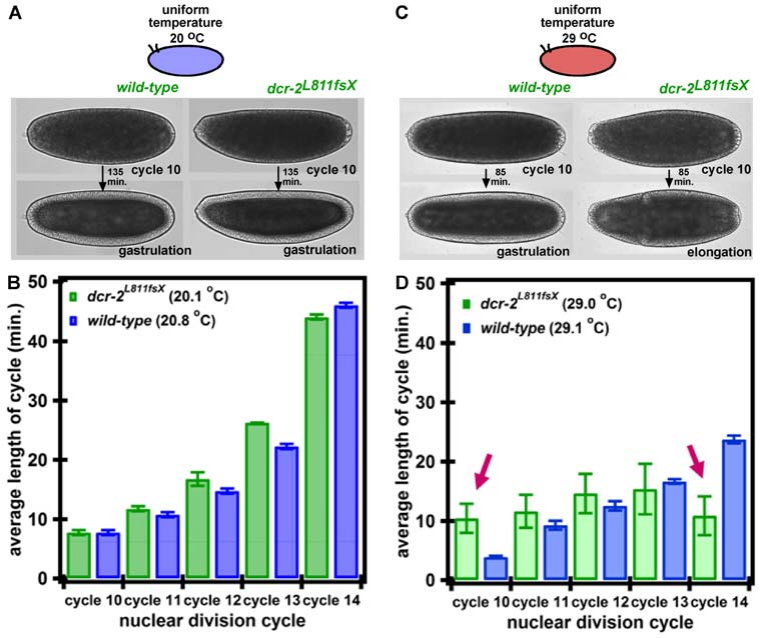
Wild-type (WT) and *dcr-2* embryos exposed to uniform 20°C and uniform 29°C. (A) *dcr-2* and WT embryos reach gastrulation at similar times at 20°C. One representation of five *dcr-2* and WT embryos is shown. (B) *dcr-2* embryos have a comparable lengthening of nuclear division cycles 10–14 to WT embryos. Nuclear division cycle lengths of five of each *dcr-2* and WT embryos are plotted. (C) *dcr-2* embryos develop more rapidly than WT embryos −85 minutes after the beginning of cycle 10, wild-type embryos reach gastrulation at 29°C, and *dcr-2^L811fsX^* embryos have reached elongation. One representation of seven *dcr-2* and five WT embryos is shown. (D) *dcr-2* embryos do not have increased length of nuclear division cycles 10–14, as do WT embryos. Average length of nuclear division cycle is shown, and error bars show the standard deviation. Averages represent six WT embryos and four *dcr-2^L811fsX^* embryos. Nuclear division cycle lengths of seven *dcr-2* and five WT embryos are plotted.

The endo-siRNA pathway presumably degrades excess mRNA synthesized at high temperatures. If *dcr-2* embryos have a decreased tolerance to differences in developmental rates, they will presumably display abnormalities in timing of development at more extreme uniform temperatures. WT embryos develop normally at a large range of temperatures. At 29°C, WT embryos have been shown to display normal gap gene expression [39], and have a more rapid, but still normal, lengthening of the cell cycle between cycles 10–14 (Figure 8C–D). In contrast, we found that *dcr-2* embryos do not have lengthened nuclear division cycles from cycles 10–14 (Figure 8C–D). Notably, cycle 10 was doubled in length in *dcr-2* embryos, and cycle 14 was considerably shortened in *dcr-2* embryos (Figure 8D). Although embryos displayed normal Hb and Eve patterning (data not shown) and did not have any obvious morphological abnormalities up to elongation, these embryos failed to reach the larval stage. *dcr-2* embryos developing at uniform 20°C had normal cell cycle lengths, comparable to those of WT embryos (Figure 8A–B), also suggesting that the endo-siRNA pathway is used to rid the embryo of excess mRNA, presumably mRNAs controlling cell cycle, at higher temperatures.

### There is Polarity to the siRNA Pathway

Up to this point, our analysis has been focused on temperature steps with the anterior half of the embryo at a higher temperature than the posterior half of the embryo. We wished to know if the endo-siRNA pathway is also essential for robust development when the anterior half is at a lower temperature than the posterior half. Surprisingly, *dcr-2* and *ago2* embryos exposed to a temperature step of the opposite orientation (anterior at 20°C and posterior at 27°C) showed normal numbers of Eve stripes, though with slightly varied positioning (Figure S5). Likewise, the profile of Hb protein expression was also less abnormal in *dcr-2* embryos exposed to this temperature step (Figure S6)–the posterior peak of Hb expression was present. These results suggest that there is an anteroposterior polarity to the robustness mechanism that is mediated by the endo-siRNA pathway.

## Discussion

Our study provides evidence that the endo-siRNA pathway, previously thought to be non-essential during development, is essential to compensate against temperature fluctuations, specifically by regulating the cell cycle in the developing *Drosophila* embryo under environmental perturbations. The endo-siRNA pathway has previously been shown to be necessary for viral immunity in *Drosophila* adults. Flock house virus (FHV) replicates more effectively in *dcr-2* embryos that have been exogenously infected, suggesting that the endo-siRNA pathway can reduce viral load in embryos [24], [25]. It is possible that increased viral load in mutant embryos generates stress on developmental programs, accounting for our results. However, we do not favor this interpretation. No endogenous FHV was detected in our WT embryos, or in *dcr-2* and *ago2* embryos (Marques, J. and Carthew, R.W., unpublished data). Although a persistent *Drosophila* virus (Nora virus) [45] was detectable in all embryos, its expression level was not significantly enhanced in *dcr-2* mutant embryos under our experimental temperature step conditions (data not shown).

In *dcr-2* embryos exposed to the temperature step, severely abnormal segmentation was observed during cycle 14, in the expression of Eve, and at the larval stage in the exoskeletal cuticle. These results suggest that the maternal morphogens and early zygotic gap genes, which set up the body plan of the embryo, may be directly regulated by endo-siRNAs. Recent results monitoring the dynamics of the Bicoid gradient suggest regulation of early patterning at the level of mRNA [12]. While we cannot rule out direct regulation of segmentation by endo-siRNAs, the anterior domain of Hb expression in *dcr-2* embryos was largely normal in seven of eight embryos. Future experiments characterizing the expression pattern of all maternal and gap genes, especially those expressed posteriorly, in *dcr-2* embryos exposed to the temperature step will delineate potential direct regulation of the segmentation network by endo-siRNAS.

A more striking defect in Hb expression was the nearly complete loss of posterior expression. This lack of expression of the posterior stripe of Hb in *dcr-2* embryos exposed to a temperature step, in combination with the highly underdeveloped posterior expression of both Eve and later larval segmentation, suggests that the difference in rate of development in the warm, anterior (faster development) and cool, posterior (slower development) halves of the embryo was not being compensated for, as it was in WT embryos observed previously [10]. In fact, when a greater difference in the rate of development is induced in WT embryos by placing them in a temperature step with a larger difference in temperature (anterior at 27°C and posterior at 17°C), a larval phenotype was observed which is similar to that of the *dcr-2* mutant embryos exposed to a temperature step with a smaller difference in temperature. We therefore propose that the underdevelopment and lack of Hb and Eve expression in the posterior half of *dcr-2* embryos is due to slower progression of cell cycle in the posterior (cool) half of the embryo relative to the anterior (warm) half of the embryo. When *dcr-2* embryos were placed at uniform high temperature (29°C), nuclear division cycles 10–14 did not lengthen as they did in WT embryos or *dcr-2* embryos at uniform low temperature (20°C). This result suggests that *dcr-2* embryos did not regulate cell cycle properly at uniform high temperatures, and is in agreement with the lack of Hb and Eve expression observed in the posterior, cool half of *dcr-2* embryos exposed to the temperature step.

Given the absence of lengthening of the cell cycle in *dcr-2* embryos at uniform high temperature, we hypothesize a model in which the endo-siRNA pathway generates endo-siRNAs that act upon regulators of the cell cycle (Figure S7). Previous studies suggest that degradation of maternal cyclins causes the lengthening of the cell cycle during cycles 10 to 13 [46], [47]. When the maternal dose of Cyclin B is reduced, cycles 10–13 are more delayed than in WT embryos [47]. Conversely, in embryos with extra copies of CYCLIN B, nuclear division cycles 10–13 did not lengthen as much as wild-type embryos [46]. In addition to the degradation of maternally provided cyclins, depletion of factors involved in DNA replication have also been shown to have an effect on cell cycle length during cycles 10–13. *grapes* or *Mei-41* embryos continued the rapid nuclear division cycles from cycles 10–13 [48], [49]. While maternal cyclins and factors involved in DNA replication are implicated in cell cycle lengthening prior to cycle 14, the G2_14_ arrest during cycle 14 is presumably due to degradation of maternal CDC25^string^. Mutations or modifications of the copy number in CYCLIN B, GRAPES, and MEI-41 led to a lack of cell cycle lengthening from cycles 10–13, and modifications in the copy number of CDC25^string^ resulted in an absence of cycle 14 lengthening [50], which is what we observed in our *dcr-2* embryos at 29°C. Additionally, when zygotic transcription was blocked prior to cycle 6, CDC25^string^ was stabilized, suggesting that a zygotic factor (e.g. a short RNA) may be responsible for the degradation of CDC25^string^.

However, there are notable discrepancies between the observed phenotypes presented in our data and previous results. For example, *grapes* embryos do not cellularize or gastrulate [51], unlike *dcr-2* embryos at 29°C. We also note that in our experiments with *dcr-2* embryos at 29°C, cycle 10 was double its length in WT embryos at 29°C; whereas, cycles 11–13 did not lengthen. This pattern of lengths of cell cycles was not observed in embryos mutant in cell cycle factors.

Recently, a number of studies have identified a large and diverse collection of endo-siRNAs in *Drosophila* [29]–[31]. These correspond to sequences derived from transposable elements and also to a variety of protein-coding genes. The functions of these endo-siRNAs are for the most part, unknown. We speculate that some endo-siRNAs function to generate robustness. Because the endo-siRNA pathway represses gene expression, it is simplest to hypothesize that the endo-siRNA pathway attenuates overactive or accelerated gene expression that is occurring in the warmer anterior end of an embryo exposed to a temperature step. In doing so, it would tune down anterior gene expression and thereby allow the slower posterior processes to catch up. In the absence of endo-siRNAs, the anterior patterning would proceed unchecked, leading to an expansion of the anterior pattern, as we observed. This could presumably occur at the expense of the posterior pattern, which would be inhibited.

A number of current models of robustness in development focus exclusively on regulation in DNA and protein networks, although a recent model suggested that small RNAs could aid in sharpening borders of zygotic gene expression [52]. The results presented here imply that regulation at the RNA level is necessary to describe robustness of development in *Drosophila* and, potentially, other organisms. In addition, results presented herein shed light on the components involved in temperature compensation, and suggest that the endo-siRNA pathway may be a general network for buffering against fluctuations in mRNA concentrations due to temperature variations.

## Materials and Methods

### Generation of Homozygous dcr-2 Null Mutant Stocks

The FLP/FRT system was used to generate females with homozygous *dcr-2^R416x^* mutant germ cells [20]. *y w ey FLP; FRT 42D dcr-2*/*CyO* females were crossed to *hsFLP; FRT 42D P[ovoD1]* males, and mitotic recombination was induced as previously described [53]. Females and males from this cross were mated, and embryos were used for the experiments described.

### Detection of Eve and Hb Expression

Embryos were fixed in 3% formaldehyde in PEM buffer immediately after removal from the temperature step. Eve and Hb expression were detected by fluorescent immunostaining using anti-Eve (rabbit monoclonal) [37] and anti-Hb (mouse monoclonal 1G10) [54] antibodies for primary detection, and goat anti-rabbit IgG (H+L) AlexaFluor 488 or AlexaFluor 594 and goat anti-mouse IgG (H+L) AlexaFluor 488 conjugated secondary antibody (Molecular Probes) for secondary detection.

### Image Acquisition and Quantification of Eve Expression

Images were acquired using a Leica DMI6000 inverted microscope with a 20x 0.7 NA objective and a cooled ORCA ERG 1394 CCD camera (12-bit, 1344×1024 resolution, Hamamatsu Photonics, K.K.). Eve expression was quantified by measuring the intensity as a function of the length of the embryo using MetaMorph® Imaging System (Universal Imaging Corp).

### Cuticle Preparation

Cuticles of larvae were prepared as described previously [55] in Hoyer's media. The cuticle preparations were visualized by DIC optics using a 10x 0.3 NA objective on a Leica DM IRE2 inverted microscope and a cooled ORCA ERG 1394 CCD camera (12-bit, 1344×1024 resolution, Hamamatsu Photonics, K.K.).

## Supporting Information

Figure S1 Expression pattern of Even-skipped (Eve) in *dcr-2^R416x^*, *dcr-2^L811fsX^*, and *ago2^414^* mutant embryos developed at a uniform temperature. (A–C) Eve expression is normal in *dcr-2^R416x^* embryos allowed to develop at a uniform temperature of 20°C (A), 24°C (B), or 27°C (C). (D–F) Eve expression is normal in *dcr-2^L811fsX^* embryos allowed to develop at a uniform temperature of 20°C (D), 24°C (E), or 27°C (F). (G–I) Eve expression is normal in *ago2^414^* embryos allowed to develop at a uniform temperature of 20°C (G), 24°C (H), or 27°C (I).
(1.16 MB TIF)

Figure S2 Cuticle preparations of *dcr-2^R416x^* and *dcr-2^L811fsX^* larvae developed at uniform temperature (24°C) or from embryos developed in a temperature step for the first 200 minutes of development and then allowed to reach larval stage at uniform temperature (24°C). (A,C) All five *dcr-2^R416x^* and all five *dcr-2^L811fsX^* larvae that developed at 24°C appear normal. (B,D) All five *dcr-2^R416x^* larvae and all three *dcr-2^L811fsX^* larvae from embryos that were exposed to the temperature step appear abnormal. (B) In *dcr-2^R416x^* mutants, four of five larvae had between four to six denticle belts. One larva had eight denticle belts, but with abnormal spacing between belts two and three. (D) In *dcr-2^L811fsX^* mutants, two of three larvae hatched but had only four denticle belts. One larva failed to hatch and had only one clear belt. Total larvae numbers include larvae shown in the main text.
(4.22 MB TIF)

Figure S3 Cuticle preparations of *ago2^414^* larvae developed at uniform temperature (24°C) or from embryos developed in a temperature step for the first 200 minutes of development and then allowed to reach larval stage at uniform temperature (24°C). (A) All four *ago2^414^* larvae developed at 24°C appear normal. (B) Two out of three *ago2^414^* larvae from embryos that were exposed to the temperature step appear abnormal, having four or six denticle belts. One out of three larvae had all eight denticle belts. Total larvae numbers include larvae shown in the main text.
(1.94 MB TIF)

Figure S4. Cuticles of *dcr-2^L811fsX^; P[w^+^; dcr-2]* embryos that were developed in a temperature step for 200 minutes and then allowed to grow to larval stage at uniform 24°C. Six out of eight larvae developed normally. One larva was missing two denticle belts, and the other larvae had abnormal position of one denticle belt. Total larvae numbers include larvae shown in the main text.
(2.17 MB TIF)

Figure S5 Expression pattern of Even-skipped (Eve) in *dcr-2^R416x^* and *ago2^414^* mutant embryos developed in a temperature step with anterior at 20°C and posterior at 27°C. (A) All three *dcr-2^R416x^* embryos had the correct number but of Eve stripes, but one *dcr-2^R416x^* embryo had slightly abnormal position of Eve stripes. (B) Both *ago2^414^* embryos had the correct number of Eve stripes. These results suggest polarity to the robustness.
(0.38 MB TIF)

Figure S6 A normal expression pattern of Hunchback (Hb) is observed in *dcr-2^R416x^* mutant embryos developed in a temperature step with anterior at 20°C and posterior at 27°C.
(0.35 MB TIF)

Figure S7 Regulation of cycles 2–14 in the *Drosophila* embryo. (A) During cycles 2–7, all maternal cyclins and Cdc2 are in excess. The nuclear divisions proceed rapidly, and are not limited by the concentration of cyclins or Cdc2. During nuclear division cycles 8–13, Cyclin A and Cyclin B are degraded, presumably by an increase in a nuclear factor. The translation of additional Cyclin A and Cyclin B protein becomes rate limiting, and nuclear division cycles lengthen progressively from cycles 8–13. During nuclear division cycle 14, maternal *cdc25^string^* is degraded, and transcription of zygotic *cdc25^string^* becomes rate limiting, causing a cell cycle arrest in G2_14_. (B) Molecular components that drive mitosis in the cell cycle.
(1.29 MB TIF)
