# Supplementary figures and images for: The Endo-siRNA Pathway Is Essential for Robust Development of the Drosophila Embryo
Source: PLoS One. 2009 Oct 23;4(10):e7576. doi: 10.1371/journal.pone.0007576 (PMC2761733; doi:10.1371/journal.pone.0007576)

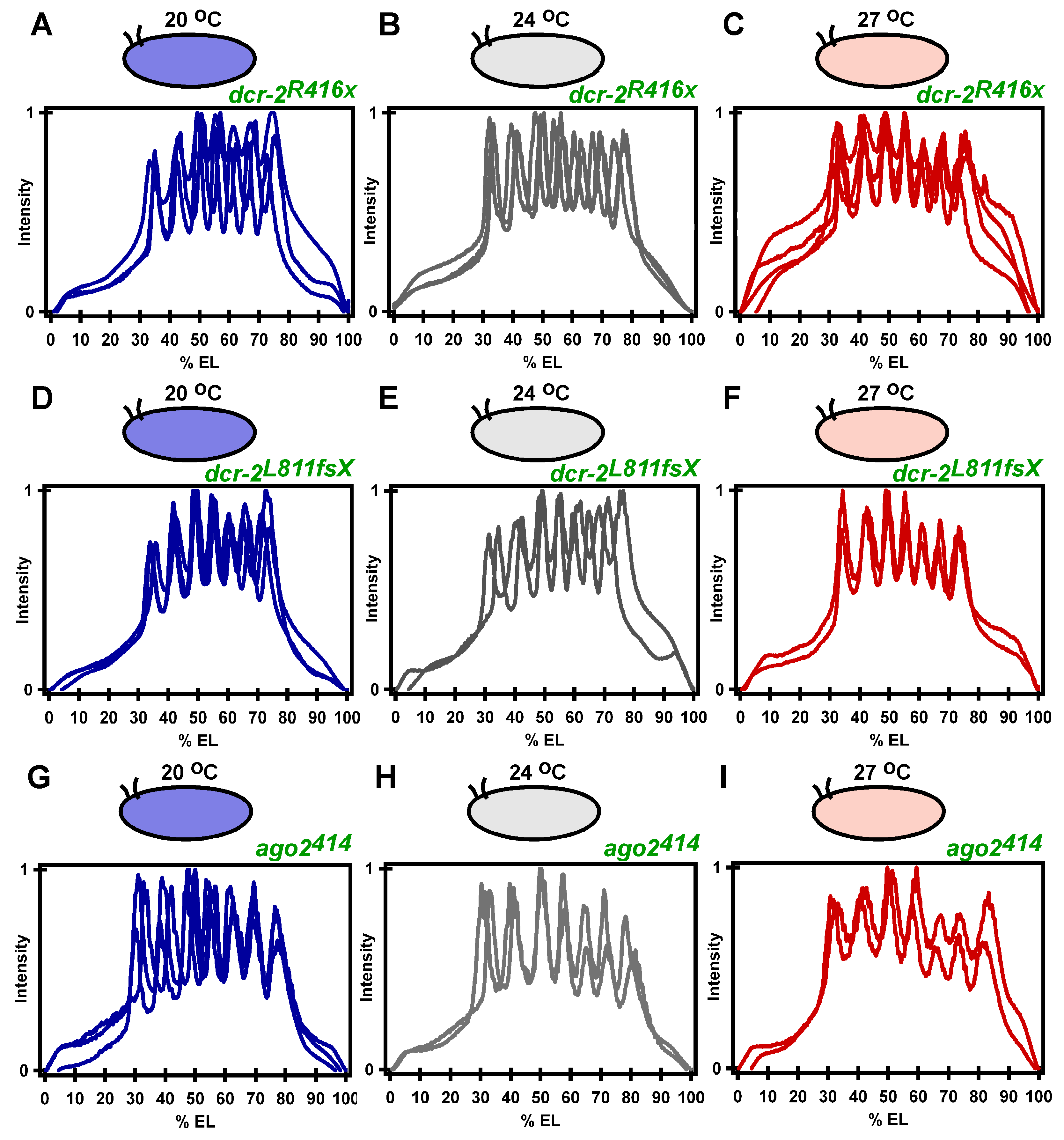

Supplement: Figure S1 — Expression pattern of Even-skipped (Eve) in dcr-2R416x, dcr-2L811fsX, and ago2414 mutant embryos developed at a uniform temperature. (A–C) Eve expression is normal in dcr-2R416x embryos allowed to develop at a uniform temperature of 20°C (A), 24°C (B), or 27°C (C). (D–F) Eve expression is normal in dcr-2L811fsX embryos allowed to develop at a uniform temperature of 20°C (D), 24°C (E), or 27°C (F). (G–I) Eve expression is normal in ago2414 embryos allowed to develop at a uniform temperature of 20°C (G), 24°C (H), or 27°C (I). (1.16 MB TIF) [file pone.0007576.s001.tif]

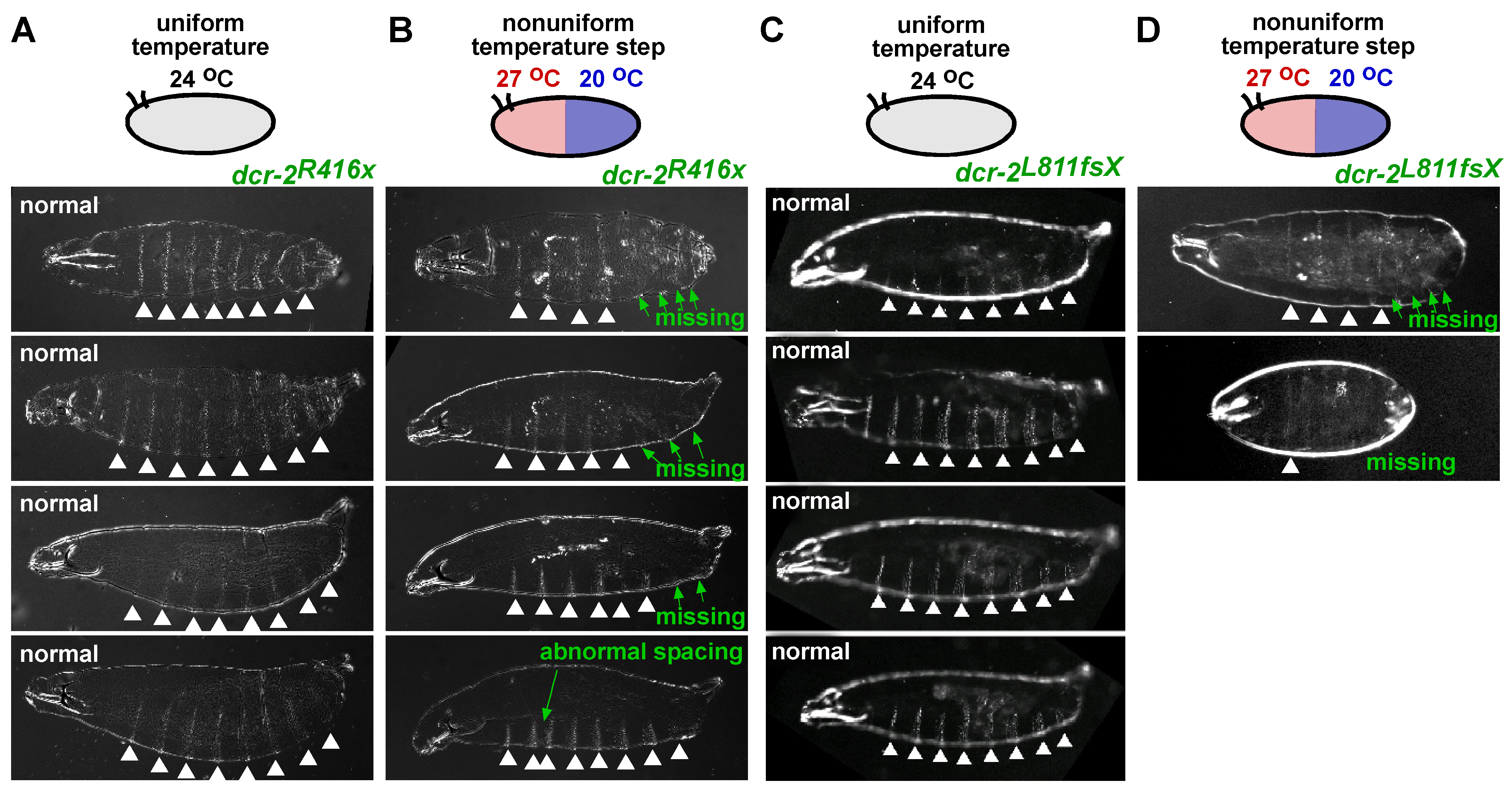

Supplement: Figure S2 — Cuticle preparations of dcr-2R416x and dcr-2L811fsX larvae developed at uniform temperature (24°C) or from embryos developed in a temperature step for the first 200 minutes of development and then allowed to reach larval stage at uniform temperature (24°C). (A,C) All five dcr-2R416x and all five dcr-2L811fsX larvae that developed at 24°C appear normal. (B,D) All five dcr-2R416x larvae and all three dcr-2L811fsX larvae from embryos that were exposed to the temperature step appear abnormal. (B) In dcr-2R416x mutants, four of five larvae had between four to six denticle belts. One larva had eight denticle belts, but with abnormal spacing between belts two and three. (D) In dcr-2L811fsX mutants, two of three larvae hatched but had only four denticle belts. One larva failed to hatch and had only one clear belt. Total larvae numbers include larvae shown in the main text. (4.22 MB TIF) [file pone.0007576.s002.tif]

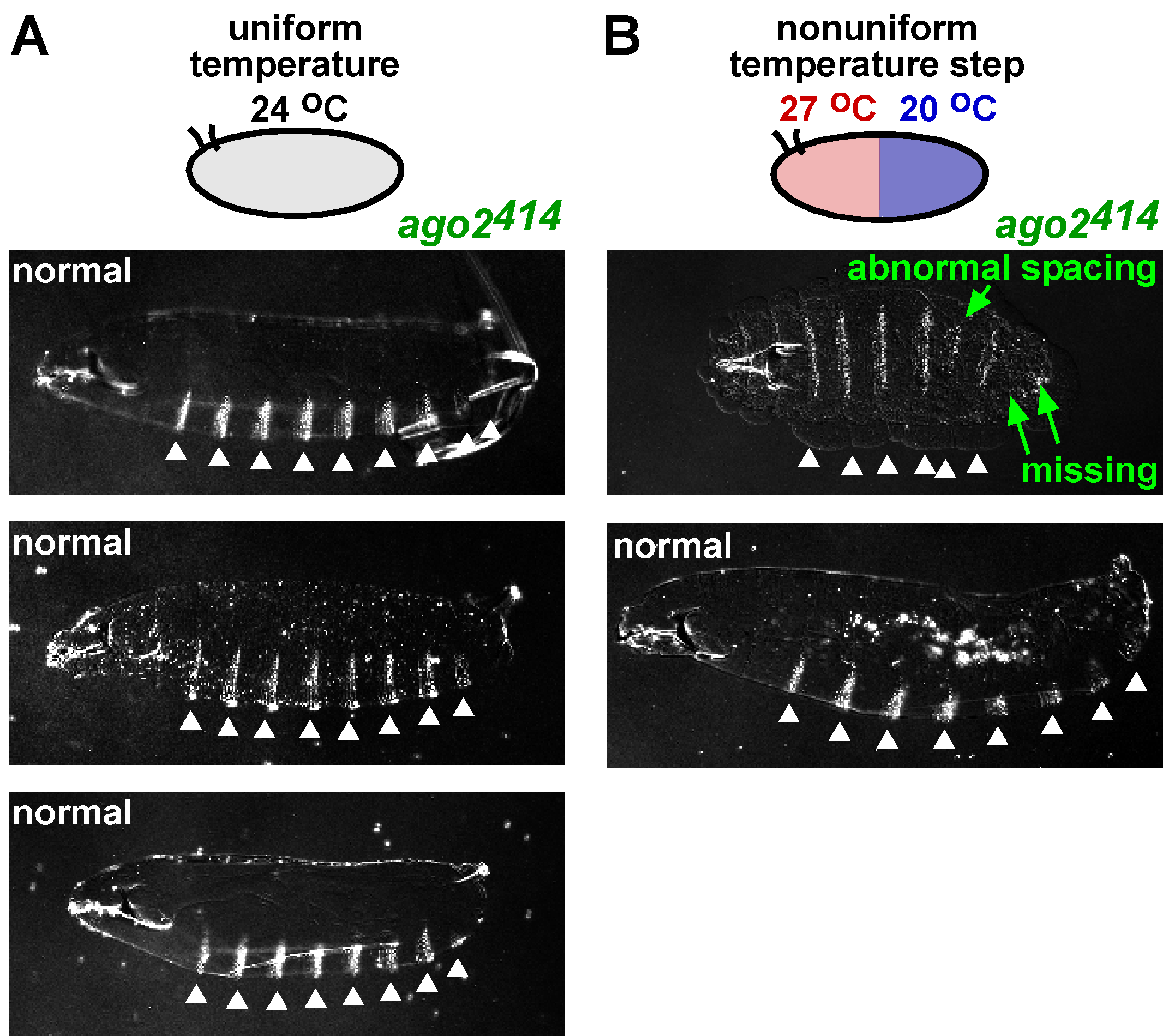

Supplement: Figure S3 — Cuticle preparations of ago2414 larvae developed at uniform temperature (24°C) or from embryos developed in a temperature step for the first 200 minutes of development and then allowed to reach larval stage at uniform temperature (24°C). (A) All four ago2414 larvae developed at 24°C appear normal. (B) Two out of three ago2414 larvae from embryos that were exposed to the temperature step appear abnormal, having four or six denticle belts. One out of three larvae had all eight denticle belts. Total larvae numbers include larvae shown in the main text. (1.94 MB TIF) [file pone.0007576.s003.tif]

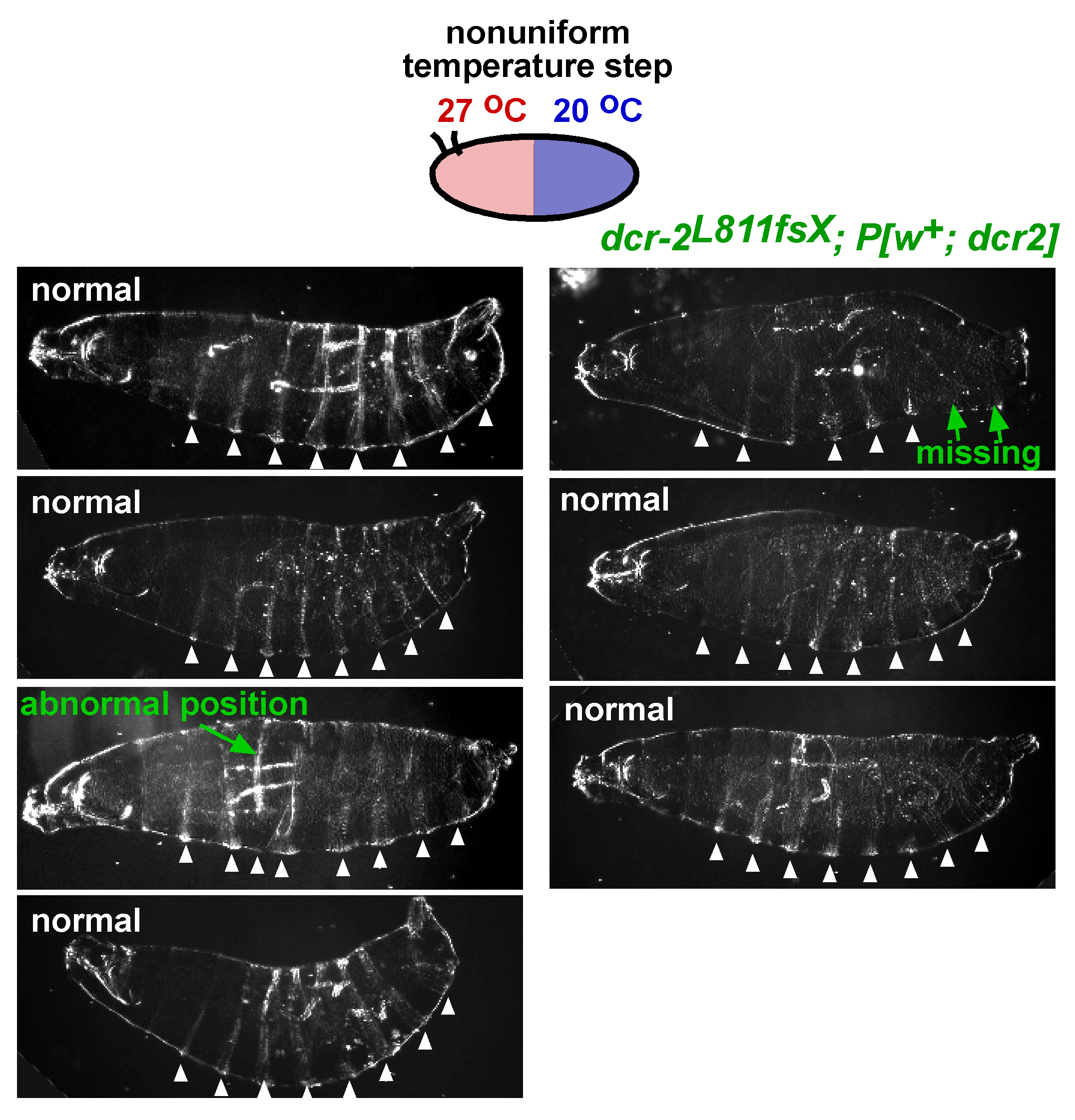

Supplement: Figure S4. — Cuticles of dcr-2L811fsX; P[w+; dcr-2] embryos that were developed in a temperature step for 200 minutes and then allowed to grow to larval stage at uniform 24°C. Six out of eight larvae developed normally. One larva was missing two denticle belts, and the other larvae had abnormal position of one denticle belt. Total larvae numbers include larvae shown in the main text. (2.17 MB TIF) [file pone.0007576.s004.tif]

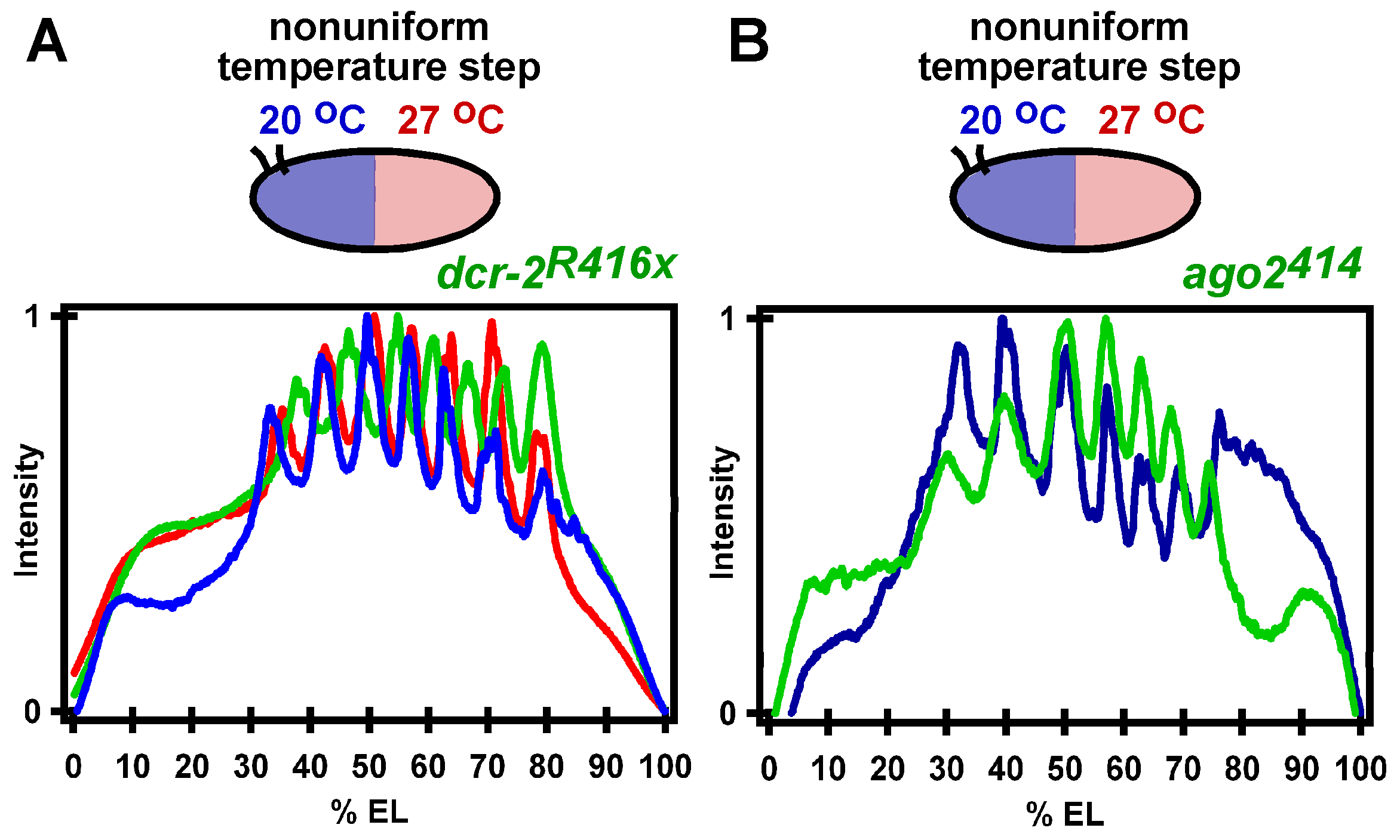

Supplement: Figure S5 — Expression pattern of Even-skipped (Eve) in dcr-2R416x and ago2414 mutant embryos developed in a temperature step with anterior at 20°C and posterior at 27°C. (A) All three dcr-2R416x embryos had the correct number but of Eve stripes, but one dcr-2R416x embryo had slightly abnormal position of Eve stripes. (B) Both ago2414 embryos had the correct number of Eve stripes. These results suggest polarity to the robustness. (0.38 MB TIF) [file pone.0007576.s005.tif]

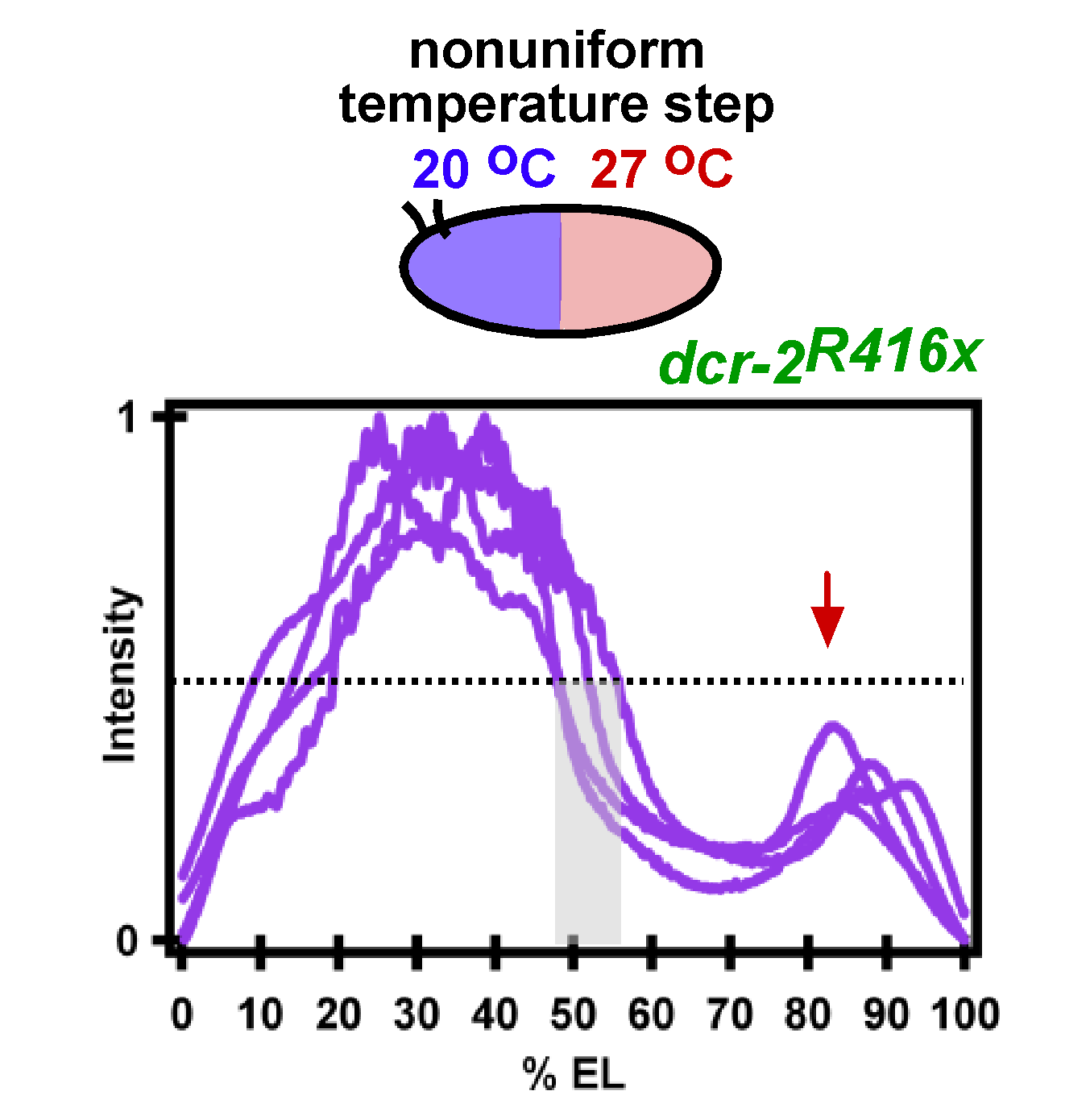

Supplement: Figure S6 — A normal expression pattern of Hunchback (Hb) is observed in dcr-2R416x mutant embryos developed in a temperature step with anterior at 20°C and posterior at 27°C. (0.35 MB TIF) [file pone.0007576.s006.tif]

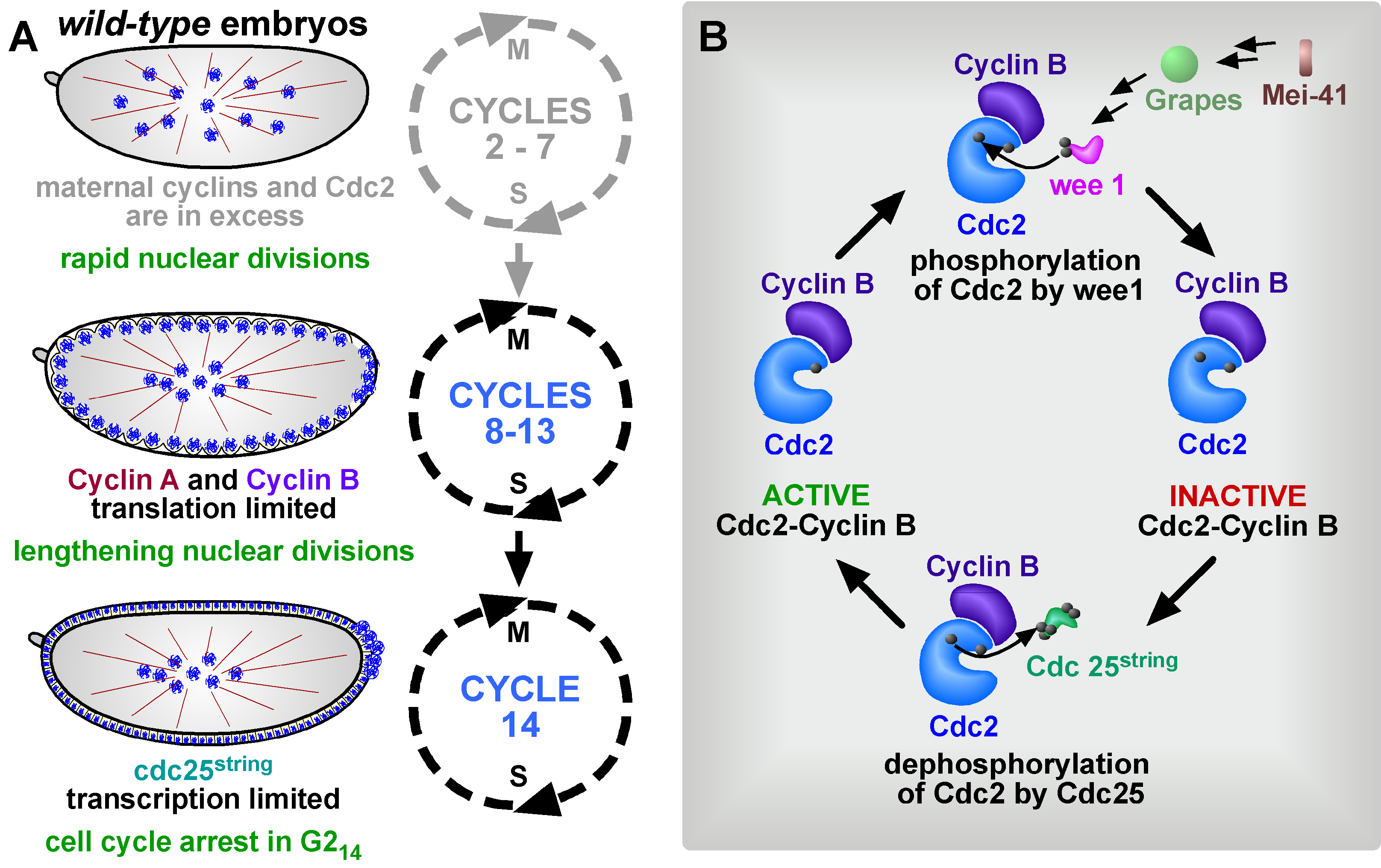

Supplement: Figure S7 — Regulation of cycles 2–14 in the Drosophila embryo. (A) During cycles 2–7, all maternal cyclins and Cdc2 are in excess. The nuclear divisions proceed rapidly, and are not limited by the concentration of cyclins or Cdc2. During nuclear division cycles 8–13, Cyclin A and Cyclin B are degraded, presumably by an increase in a nuclear factor. The translation of additional Cyclin A and Cyclin B protein becomes rate limiting, and nuclear division cycles lengthen progressively from cycles 8–13. During nuclear division cycle 14, maternal cdc25string is degraded, and transcription of zygotic cdc25string becomes rate limiting, causing a cell cycle arrest in G214. (B) Molecular components that drive mitosis in the cell cycle. (1.29 MB TIF) [file pone.0007576.s007.tif]
